# Fourier Ring Correlation and Anisotropic Kernel Density Estimation Improve Deep Learning Based SMLM Reconstruction of Microtubules

**DOI:** 10.3389/fbinf.2021.752788

**Published:** 2021-10-15

**Authors:** Andreas Berberich, Andreas Kurz, Sebastian Reinhard, Torsten Johann Paul, Paul Ray Burd, Markus Sauer, Philip Kollmannsberger

**Affiliations:** ^1^ Center for Computational and Theoretical Biology, University of Wuerzburg, Wuerzburg, Germany; ^2^ Department of Biotechnology and Biophysics, University of Wuerzburg, Wuerzburg, Germany; ^3^ Institute for Theoretical Physics and Astrophysics, University of Wuerzburg, Wuerzburg, Germany

**Keywords:** *d*STORM, deep learning–artificial neural network (DL-ANN), single molecule localization microscopy, microtubule cytoskeleton, super-resolution

## Abstract

Single-molecule super-resolution microscopy (SMLM) techniques like *d*STORM can reveal biological structures down to the nanometer scale. The achievable resolution is not only defined by the localization precision of individual fluorescent molecules, but also by their density, which becomes a limiting factor e.g., in expansion microscopy. Artificial deep neural networks can learn to reconstruct dense super-resolved structures such as microtubules from a sparse, noisy set of data points. This approach requires a robust method to assess the quality of a predicted density image and to quantitatively compare it to a ground truth image. Such a quality measure needs to be differentiable to be applied as loss function in deep learning. We developed a new trainable quality measure based on Fourier Ring Correlation (FRC) and used it to train deep neural networks to map a small number of sampling points to an underlying density. Smooth ground truth images of microtubules were generated from localization coordinates using an anisotropic Gaussian kernel density estimator. We show that the FRC criterion ideally complements the existing state-of-the-art multiscale structural similarity index, since both are interpretable and there is no trade-off between them during optimization. The TensorFlow implementation of our FRC metric can easily be integrated into existing deep learning workflows.

## Introduction

Single-molecule localization microscopy (SMLM) can overcome the diffraction barrier in fluorescence microscopy by stretching the activation of fluorophores over time. To achieve this, individual non-overlapping active emitters are localized with a precision of a few nanometers, limited only by the number of photons acquired and the noise (van de Linde et al., 2011). The trade-off in SMLM is the acquisition time required to obtain enough localizations to reconstruct a dense super-resolved image. New deep learning-based fitting algorithms can reconstruct localizations from raw frames at higher densities (Nehme et al., 2018; Speiser et al., 2021). This allows for shorter acquisition times by increasing the number of blinking fluorophores in each frame. In some cases, however, the density of localizations is inherently limited, for example due to unstable photodyes or low emitter density in expanded samples. The density of localizations limits the resolution of SMLM independent of localization precision, since no structures at a length scale smaller than two emitter distances can be resolved according to the Nyquist limit.

Applications of deep convolutional neural networks to SMLM have so far mainly been for fitting: using raw diffraction-limited frames as input, trained deep networks predict localization coordinates (Zelger et al., 2018), super-resolved images (Nehme et al., 2018), or images with localization coordinates encoded in the pixel values (Speiser et al., 2021). By learning non-linear mappings from intensity distributions to point coordinates, the sparsity requirements needed for accurate Gaussian fitting can be relaxed and much higher localization densities can be imaged, thus reducing the necessary measuring time. In fluorescence microscopy in general, deep learning has many other applications, including denoising and image restoration (Weigert et al., 2018), classification, and segmentation. Most of these applications are image-to-image tasks, i.e., the network takes images as input and generates denoised images or segmentation labels as output.

For image-to-image tasks like denoising and segmentation, the *U-Net* architecture introduced by (Ronnebergeret al., 2015) is considered state-of-the-art. It consists of an autoencoder-like convolutional network with additional skip connections between the down- and upsampling part. This way, a high-level feature-based representation is efficiently combined with spatial information. To train a U-Net, the generated image or label map is compared to a ground truth image using various image-based metrics (Zhao et al., 2017). The classical metric in image-to-image tasks is the L_2_ loss, corresponding to the pixel-wise mean squared error between output and target image. L_2_ is common standard but causes artifacts as it does not penalize small errors. The structural similarity index measure (SSIM) is a good alternative as it takes the properties of the human perceptive system into account. It shows best results when combined with the absolute pixel-wise error or L_1_ loss to prevent an intensity offset (Zhao et al., 2017). Alternatively, the loss function can be learned by optimizing the image generator against a second network (discriminator) that tries to discriminate ground truth images from those generated by the U-Net in an approach called “conditional generative adversarial network” or *cGAN* (Isola et al., 2017). While this architecture can learn to generate surprisingly realistic-looking images, the authors note that it is not suitable for segmentation due to its tendency to generate plausible-looking but non-existing structures in images.

To reconstruct dense SMLM images from sparse subsets of localization, ANNA-PALM by (Ouyang et al., 2018) elegantly combines the *pix2pix* cGAN architecture from (Isola et al., 2017) with a consistency check against low-resolution images to overcome the limitations of generative networks. In addition, multiscale SSIM and L_1_ loss as described in (Zhao et al., 2017) are used for training the U-Net generator. The generator-discriminator loss by itself cannot be interpreted as measure of prediction accuracy, as the two networks depend on each other. During supervised training, there are ground truth images that can serve as target to compare the prediction to the ground truth and to determine the error, but when applying the trained network to new images, this information is not available. To solve this problem, a comparison with low-resolution wide-field images is performed in (Ouyang et al., 2018) in cases where such images are available.

Due to the stochastic blinking during the measurement, the SMLM imaging process can be interpreted as sampling from an underlying fluorophore-labeled density. This sampling contains errors due to mislabeling, photobleaching, and post-processing. Hence, one goal for each SMLM method is to estimate the real underlying fluorophore distribution from a measured error-prone sample. In some cases, the precise coordinates of individual emitters are relevant, for example when looking at the relative arrangements of discrete, isolated labeled molecules. In most cases, however, the individual locations are of secondary interest, and the reconstruction of the underlying density is the central goal. This is the case for example when imaging continuous structures in the cell, for example, cytoskeletal filaments.

Localization coordinates can be visualized in different ways to give an impression of the underlying density. When represented as 2D histogram where each pixel contains the number of localizations detected within the area of the pixel, blurring each localization with a Gaussian kernel with a variance sigma corresponding to the localization uncertainty can give a more accurate impression of density. A single value for sigma based on the average localization uncertainty of the entire image is an efficient approximation (R. P. J. Nieuwenhuizen et al., 2014). This Gaussian filter is an example of a kernel density estimate (KDE) with a constant (non-adaptive) kernel width sigma. More elaborate versions of KDE use adaptive kernels, for example with a sigma proportional to the density of localizations in the region of the image. Some interesting aspects of density estimation from discrete localizations are described in (Rees et al., 2012). Going one step further, the kernel for density estimation could be made anisotropic. In different context, adaptive anisotropic kernel density estimates have been used by (Hensen et al., 2009) for improving configurational entropies of macromolecules, or by (Ronneberger et al., 2015) for human motion capture. The use of adaptive anisotropic KDE for density estimation in SMLM localization data was demonstrated by (Chen et al., 2014). They used anisotropic Gaussian kernels where the covariance is a function of the surrounding density of points and show that thresholding the estimated density results in a better segmentation of subcellular structures compared to conventional Gaussian rendering.

Estimating the resolution in a single image is not easily possible, but when two images of the same structure are available, their resolution can be estimated using Fourier Ring Correlation (FRC). The 2D cross correlation of the two images is calculated, and the intensity in the Fourier transformed correlation image is summed up and binned by frequency. The resulting curve shows how much the signal in the two images is correlated as a function of frequency, or correspondingly, length scale. If the images are dominated by uncorrelated random noise beyond a certain frequency or below a certain length scale, then these length scales cannot be resolved. FRC was originally developed for electron cryomicroscopy, where two independent images each using one half of the information are compared. It can easily be applied to SMLM, since it is possible to reconstruct two subimages using one half of the localizations each (Robert P. J. Nieuwenhuizen et al., 2013). FRC is also used for image volume reconstruction, where adjacent slices in a volume can be compared against each other using the integral of the FRC (Preusser et al., 2021). FRC has recently been shown to be useful to improve and monitor image restoration and deconvolution, and can also be applied to single images by constructing different subsamplings (Koho et al., 2019). It was noted in (Legant et al., 2016) that the result cannot always be interpreted as a measure for image resolution, and that care must be taken when two different types of images are compared. FRC can vary within images depending on the content, and local FRC maps can be used to compare super-resolved images to wide-field images (Culley et al., 2018). A suitable quantification of SMLM resolution remains challenging and is an active field of research (Cohen et al., 2019; Descloux et al., 2019). A robust quality measure of reconstructed images is a key requirement to assess image reconstruction methods, and there is a general interest to develop robust quality measures for SMLM images and to integrate them into trainable image reconstruction workflows. FRC as an established measure in the SMLM field is a promising candidate for such a measure but using it for deep learning would require it to be available as differentiable loss function.

Here, we present a deep learning approach to reconstruct density estimates for microtubules from small subsets of localizations. We show how the preprocessing of ground truth images can be improved by using an anisotropic kernel density estimate. We then introduce a new loss function based on a modified FRC criterion and implement it as differentiable function that can be used for training deep neural networks. In combination, this can help to make deep learning based SMLM density reconstruction easier to interpret. The FRC loss is compared to the multiscale structural similarity index (MSSIM) by training a U-Net with different combinations of loss functions to reconstruct microtubules. As ground truth, we use conventional Gaussian rendered histograms and density estimates based on anisotropic adaptive kernels. Finally, we discuss the differences of our approach to the existing state of the art (ANNA-PALM) regarding ease of use and interpretability. Our implementation is openly available on our github repository, enabling its application for trainable image reconstruction also beyond SMLM.

## Materials and Methods

### Cell Culture, Fixation, and Staining

African green monkey kidney fibroblast-like cells (COS7, Cell Lines Service GmbH, Eppelheim, #605470) were cultured in DMEM (Sigma, #D8062) containing 10% FCS (Sigma-Aldrich, #F7524), 100 U/ml penicillin and 0.1 mg/ml streptomycin (Sigma-Aldrich, #P4333) at 37°C and 5% CO_2_. Cells were grown in standard T25-culture flasks (Greiner Bio-One). Staining of tubulin filaments was performed as described earlier (van de Linde et al., 2011). COS-7 cells were permeabilized for 1–2 min and simultaneously pre-fixed with a prewarmed buffer (37°C) containing 0.3% glutaraldehyde and 0.25% Triton X-100 in Cytoskeletal Buffer. The buffer is then exchanged for preheated (37°C) 2% glutaraldehyde (in CB) and incubated for 10 min. Fixation is stopped by 100 mM glycine (in PBS) step for 5 min, and cells were washed at least 3 times 5 min with PBS. Blocking of epitopes inducing unspecific labeling was carried out by 30 min incubation with 5% BSA. Primary antibody (rabbit α-tubulin, PA5-19489, Thermo Fisher) was added at concentrations of 10 μg/ml (in 5% BSA) for 60 min at room temperature, and unspecifically bound primary antibody was removed by rinsing the sample several times with 0.05% Tween20 (in PBS) solution followed by washing with normal PBS for 3 times 5 min. Secondary antibody [F (ab’) 2 goat-anti-rabbit IgG (H + L) Alexa-647] was added at concentrations of 10 μg/ml in 5% BSA for at least 60 min at room temperature. Washing steps with tween solution and PBS were applied as described above. To maintain the labeling of both antibodies a post fixation step with 4% formaldehyde (in PBS) for 10 min was performed.

### *d*STORM Imaging

Imaging was performed on a Nikon Eclipse Ti inverted wide-field microscope using a 640 nm laser at 200 mW excitation output power, a Nikon APO TIRF 100x/1.49 oil immersion objective, adapted HILO illumination and ×22 binning resulting in a pixel size of 108 nm. In total 12 spots with microtubules were imaged, and 50.000 frames were acquired per position. Each image covers a square of about 21 × 21 μm^2^. Exposure time was set to 20 ms. Raw frames were processed with the MLE fitter in Picasso (Schnitzbauer et al., 2017) using a net gradient setting of 4,500 and drift correction.

### Anisotropic Kernel Density Estimate

Localization files generated by Picasso were spatially binned into 2D histograms with a pixel size of 5 nm. These super-resolved images were then filtered by convolution of the image *I* with a discrete filter kernel *K* such that the estimated density $f\left({\vec{x}}_{i}\right)$ of the *i-th* pixel $p_{i}$ is

$$f\left({\vec{x}}_{i}\right)=\;\frac{1}{n}\sum_{j=1}^{k}I\left({\vec{x}}_{j}\right)K\left({\vec{x}}_{i},{\vec{x}}_{j}\right),$$

where ${\vec{x}}_{i}$ is the pixel’s position in the image. The summation is performed over all *k* pixels within the kernel window placed on top of *x*_*i*_. The classical isotropic Gaussian filter corresponds to the kernel

$$K\left({\vec{x}}_{i},{\vec{x}}_{j}\right)=\;\frac{1}{2\pi b}exp\left(-\frac{{\left({\vec{x}}_{i}-{\vec{x}}_{j}\right)}^{2}}{2b^{2}}\right),$$

where *b* denotes the constant variance of the Gaussian filter. This density estimator is commonly used to render super-resolved images from localization tables (R. P. J. Nieuwenhuizen et al., 2014). It accounts for the localization uncertainty but does not consider the heterogeneity and anisotropy of the localization density. Adaptive KDE as an alternative (Rees et al., 2012) scales *b* with the density of localizations but is still isotropic. The main limitation of a Gaussian KDE, adaptive or not, is that it is not sensitive to anisotropic spatial distributions of fluorophores. For anisotropic structures like microtubule filaments, the density within the filaments becomes increasingly continuous when increasing *b*, approximating the actual filament. The edges however become more and more blurred as higher frequencies in the image are increasingly suppressed by the Gaussian kernel, since it acts as a low-pass filter.

**FIGURE 1:**
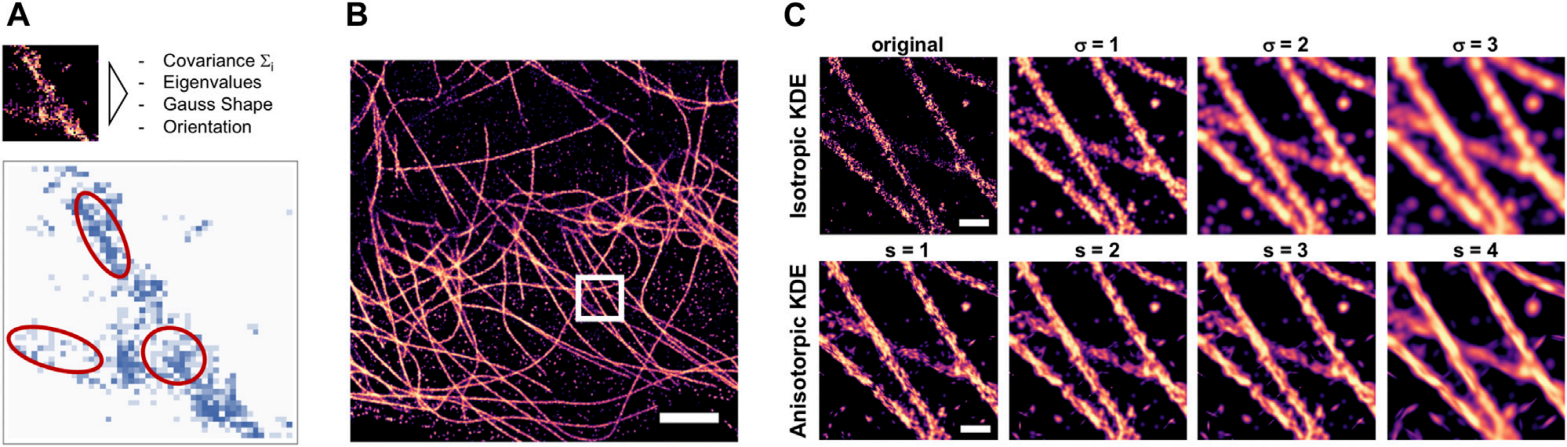
Anisotropic KDE as density estimate for filaments. **(A)** Illustration of the method as proposed by (Chen et al., 2014) but here implemented in image space; red ellipses indicate the different shapes and orientations of the 2D anisotropic Gaussian filter kernels. **(B)** Histogram rendering of a complete field of view of a reconstructed dSTORM image of microtubules in a COS7 cell, scale bar = 2 μm. **(C)** zoomed-in detail indicated by the box in b) (scale bar = 0.2 µm), top: unfiltered histogram (left) and density estimation by classical Gaussian filtering with fixed sigma of 1–3 pixels, corresponding to 5–15 nm; bottom: same region, but with anisotropic KDE applied using an adaptive multivariate Gaussian kernel scaled by a factor of 1–4 (from left to right).

The anisotropic adaptive KDE proposed by (Chen et al., 2014) uses a kernel that adapts not only its scale, but also its shape and orientation to the local distribution of localizations for each pixel. We implemented anisotropic adaptive KDE using the 2D multivariate Gaussian convolution kernel

$$K\left({\vec{x}}_{i},{\vec{x}}_{j}\right)=\;\frac{1}{\sqrt[{2}]{{\left(2\pi \right)}^{2}\left|\Sigma_{i}\right|}}\exp\left(-\frac{1}{2}{\left({\vec{x}}_{i}-{\vec{x}}_{j}\right)}^{T}\sum_{i}^{-1}\left({\vec{x}}_{i}-{\vec{x}}_{j}\right)\right),$$

where Σi is the positive definite covariance matrix that defines the properties of the kernel at position *x*_*i*_, and $\left|\Sigma_{i}\right|$ is its determinant. To adapt to the local distribution of localizations, the covariance is estimated as

$$\Sigma_{i}=\frac{1}{\tilde{I}}\sum_{j=1}^{k}\left({\vec{x}}_{j}-{\vec{\mu }}_{i}\right){\left({\vec{x}}_{j}-{\vec{\mu }}_{i}\right)}^{T}\;\mathrm{with}\;\tilde{I}=\sum_{j=1}^{k}I\left({\vec{x}}_{j}\right),$$

with ${\vec{\mu }}_{i}$ the mean intensity within the kernel window at pixel *p*_i_. Again, the summation is performed over all *k* pixels within the kernel window placed around *p*_i_. At each pixel *p*_i_, the corresponding covariance Σ_i_ shaped by the spatial intensity distribution within the filter window is calculated. The eigenvectors of Σ_i_ are perpendicular and define the orientation of the kernel, whereas its eigenvalues λ_1_ and λ_2_ define its shape. The covariance is diagonal along the main axis of the kernel, i.e., when rotated towards the direction of highest localization density. The resulting kernel is scaled by a constant factor and applied to the corresponding region of the original image. We used a constant odd window size of 11 × 11 pixel and varied the scale between 1 and 4 (Figure 1). The same approach can be used for higher dimensions, as multivariate Gaussian functions can easily be generalized to 3D, as demonstrated e.g. for spatial directional statistics simulations–see (Paul and Kollmannsberger, 2020) for an implementation in python.

### Fourier Ring Correlation Loss

Fourier Ring Correlation (FRC) measures the correlation of a pair of images as a function of spatial frequency. When applied to a pair of super-resolved images generated by dividing the list of localization coordinates in two subsamples, it can be interpreted as a measure of resolution of the full SMLM image (Robert P. J. Nieuwenhuizen et al., 2013). The two images are correlated by multiplying their Fourier transforms *F*_x_ and *F*_y_, and the FRC_xy_ is obtained by summing over concentric rings *r*_*i*_ in Fourier space:

$$\mathrm{FR}C_{xy}\left(r_{i}\right)=\frac{\sum_{r\in r_{i}}F_{x}\left(r\right)F_{y}{\left(r\right)}^{*}}{\sqrt{\sum_{r\in r_{i}}F_{x}^{2}\left(r\right)\sum_{r\in r_{i}}F_{y}^{2}{\left(r\right)}^{*}},}$$

normalized by the total intensities in each ring. The signal at a distance *r*_*i*_ from the center of the Fourier transformed images corresponds to the spatial frequency.

$$f_{i}=\frac{r_{i}}{N},$$

with *N* the number of frequency bins, or pixels in the image. The spatial frequency where the FRC falls below a value of 1/7 is defined as cut-off frequency and interpreted as resolution of the full image (Robert P. J. Nieuwenhuizen et al., 2013).

**FIGURE 2:**
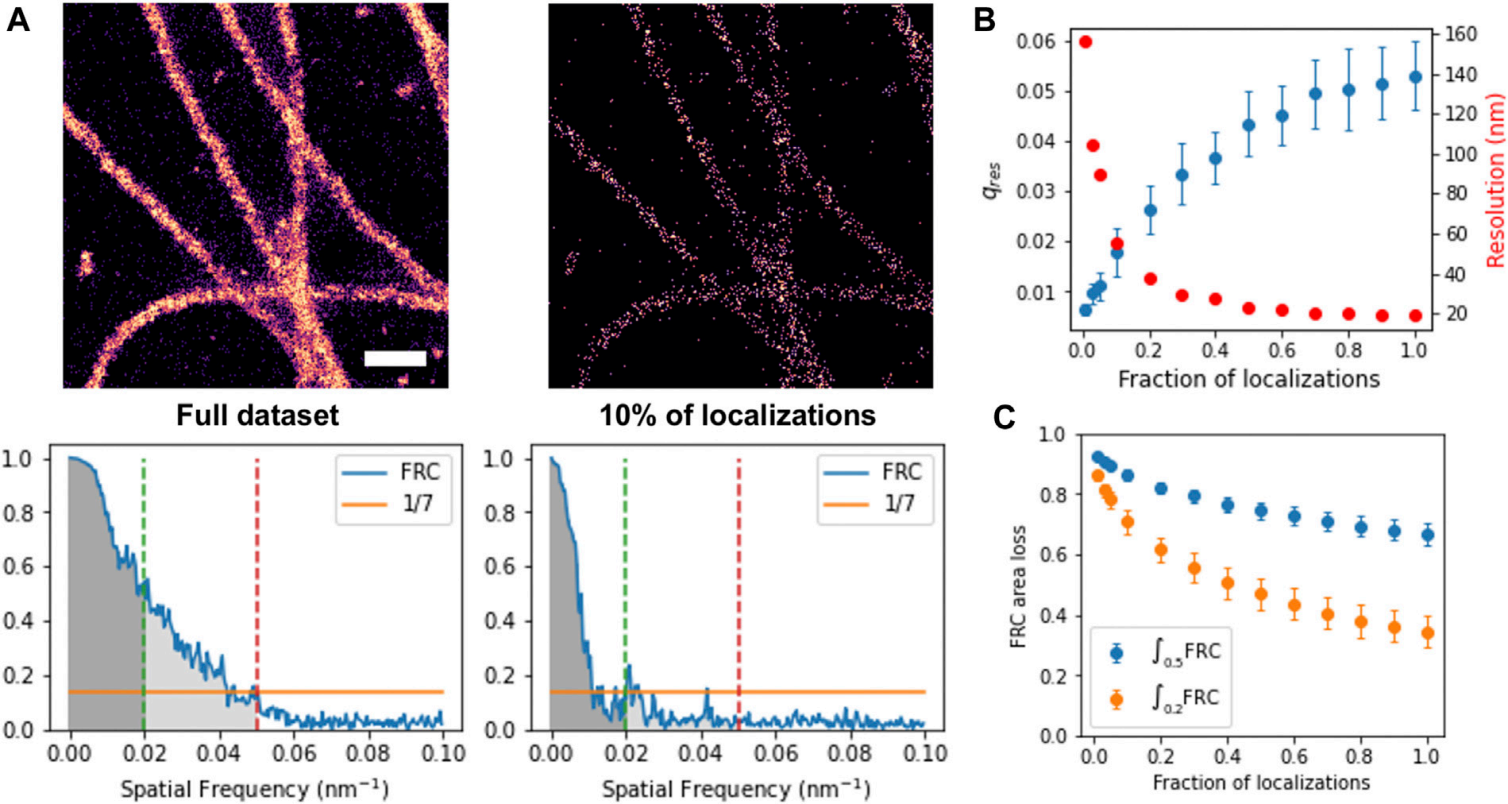
Fourier Ring Correlation to estimate image improvement with increasing density of localizations. **(A)** Histogram rendering (top) and corresponding FRC (bottom) of full and sparse set of localizations, with threshold 1/7 and area threshold 0.2 (green) and 0.5 (red) indicated. Scale bar: 300 nm, **(B)** FRC cutoff frequency (blue) and corresponding resolution (red) as function of the fraction of total localizations, **(C)** FRC area loss for a threshold of 0.2 (yellow) and 0.5 (blue) as function of the fraction of total localizations.

The value of the cut-off frequency by itself does not contain any information about the magnitude of correlation at lower frequencies. Maximizing it is thus not an ideal target for optimization (Figure 2). Instead of maximizing the cut-off frequency during optimization, we calculate the area of the FRC similar to (Preusser et al., 2021) but only up to a fixed frequency *f*′ and use it as target:

$${ℒ}_{xy}\left(f^{\prime }\right)=1-\bar{\mathrm{FR}C_{xy,f^{\prime }}}\;\mathrm{with}\;\bar{\mathrm{FR}C_{xy,f^{\prime }}}=\sum_{j=0}^{f^{\prime }}\mathrm{FR}C_{xy,f^{\prime }}\left(j\right),$$

where the summation is performed over all FRC values corresponding to the spatial frequency *j*.

We implemented the differentiable area-FRC as described above using the built-in complex multiplication, 2D-FFT and reduce_sum functions of Tensorflow2. The frequency rings are precomputed and held in GPU memory as constant masks.

### Deep Neural Network for Image Restoration

We used a 2D *U-Net*-like (Ronneberger et al., 2015) deep convolutional neural network. The details of the architecture are identical to the generator part used in the *pix2pix* cGAN (Isola et al., 2017) and in ANNA-PALM (Ouyang et al., 2018): the input image is downsampled with eight consecutive 2D convolution layers with stride = 2 and size = 4, *Leaky ReLU* activation, and increasing filter number (64-128-256-512-…-512), followed by a mirrored upsampling part with the corresponding transposed convolutions using identical stride and filter number, *ReLU* activation, and skip connections concatenating the output of the corresponding downsampling layer to the upsampling layer of the same size. The last layer is a transposed convolution with *tanh* activation and generates the final output image. The network was implemented in Tensorflow2 based on the *pix2pix* implementation in the official documentation (https://www.tensorflow.org/tutorials/generative/pix2pix) but without the discriminator part.

### Network Training

Training data were generated from localization tables produced by Picasso as follows: first, each full localization table was rendered into a 2D histogram with a pixel size of 5 nm. Isolated localizations were removed, and density was estimated either by filtering with a Gaussian blur filter of sigma = 5 nm (isotropic KDE) or by applying anisotropic KDE with window size 11 × 11 and scale factor between 1 and 4. The resulting density estimates were used as training targets. The corresponding input images were generated by rendering 2D histograms of a subset of frames using randomly selected time windows containing between 5 and 30% of the total number of localizations. Input-target image pairs were created by randomly cropping pairs of corresponding patches with a size of 750 × 750 pixels from the sparse subset images, and from the density estimates of the full dataset. From the 11 fields of view, 2 were held back for validation. During training, patches were augmented by applying continuous on-GPU rotation to prevent overfitting, and a 512 × 512 patch was cropped from the center of the rotated images. ADAM optimization with a learning rate of 2 × 10^−4^ and β_1_ = 0.5 was used to train the network for 1,000 epochs (iterations over the training set). The loss function was either area-FRC, multiscale structural similarity index, or the sum of both, as indicated. Additionally, we added a small L_1_ loss (absolute pixel-wise difference) to stabilize training, since neither MS-SIM nor FRC punish deviations in background or total intensity, which can lead to offset or inverted output images.

## Results

### Anisotropic Kernel Density Estimate

Density estimation can help to improve trainable image reconstruction algorithms that are designed to reconstruct the density from a given subset of localizations. The problem with such attempts is that the training data are available as point clouds, so the training optimizes reconstruction of discrete localization patterns rather than continuous densities. Rendered histograms of localization data contain the discrete count of localizations in each pixel, which can be non-continuous. KDE-smoothed histograms are better suited as target for trainable image reconstruction, as they do not encourage the training process to optimize for reconstructing discontinuous localization patterns, but instead for the continuous underlying density. Isotropic Gaussian KDE provides such a density estimate, but at the expense of lowering the effective resolution due to low-pass effect of Gaussian blur. We implemented an anisotropic kernel density estimation as proposed in (Chen et al., 2014) as window-based filter operation in Python (Figure 1A), and applied it to rendered histograms of localization datasets of tubulin (Figure 1B). The scale of the anisotropic KDE was systematically varied and compared to the results of conventional KDE by Gaussian filtering (Figure 1C).

The purpose of applying KDE is to obtain an estimate of the underlying true density of the labeled epitope that would be observed in the limit of perfect labeling efficiency and infinite measuring time, from the experimentally measured sample of localizations. We found that for high-density *d*STORM datasets of microtubules, the anisotropic KDE provides a better estimate of density as it does not blur the edges of the filaments (Figure 1C). We hypothesize that preprocessing of real training data with anisotropic KDE as shown in Figure 1 is an alternative approach for anisotropic structures and will result in improved reconstruction quality.

### Fourier Ring Correlation and Localization Density

Fourier Ring Correlation (FRC) can be used to measure image resolution of SMLM images by splitting the localization data in two subsets and calculating the FRC of the reconstructed histograms (Robert P. J. Nieuwenhuizen et al., 2013). Recently, FRC has been proposed to monitor the progress of image reconstruction and deconvolution methods (Koho et al., 2019). Here, we explore the potential of using FRC as target function to train deep neural networks to reconstruct the underlying density from sparse localization images. We implemented FRC in Tensorflow2 as differentiable function, as described in materials and methods. To determine how FRC depends on the localization density, we generated sparse localization datasets using a subset of frames with a defined fraction of localizations of the entire dataset. Each resulting subset of localizations was then split in two, and the FRC cut-off frequency of the corresponding rendered histograms was calculated (Figures 2A,B). When correlating sparse and dense images, the cut-off frequency cannot be directly used as resolution measure, because the already reconstructed images cannot be split in two subsets. The FRC vs. frequency plot nevertheless gives a measure for similarity between the sparse and dense images: two identical images would have FRC = 1 for all frequencies, whereas for unrelated images, FRC would be = 0 everywhere. We thus propose the integral of the FRC as new measure for reconstruction quality. To avoid the influence of spurious correlations at high frequencies, we calculate the FRC integral up to a cut-off frequency of 0.2 or 0.5 of the maximum frequency.

The dependency of our area-FRC measure is shown in Figure 2C. Here, the FRC between the reconstructed histogram of the subset and that of the full dataset was calculated and summed up to a cut-off frequency of 0.2 or 0.5. The area FRC scales similarly to the original FRC, and shows a stronger dependency on fraction of localizations when only using lower frequencies up to 0.2. For comparison, we also calculated the area FRC in the classical way, i.e., by splitting the localization data in two and summing up the FRC between the two sub-histograms (Figure 2B). As can be seen, the dependency is qualitatively similar. In summary, we conclude that the area of the FRC between the sparse and the full density SMLM image can be interpreted as a measure for the similarity between the two images. Consequently, by comparing a reconstructed density image to the true density image, one could monitor the quality of the reconstruction and thus the progress of a trainable reconstruction algorithm.

### Training Neural Networks With FRC Loss

**FIGURE 3:**
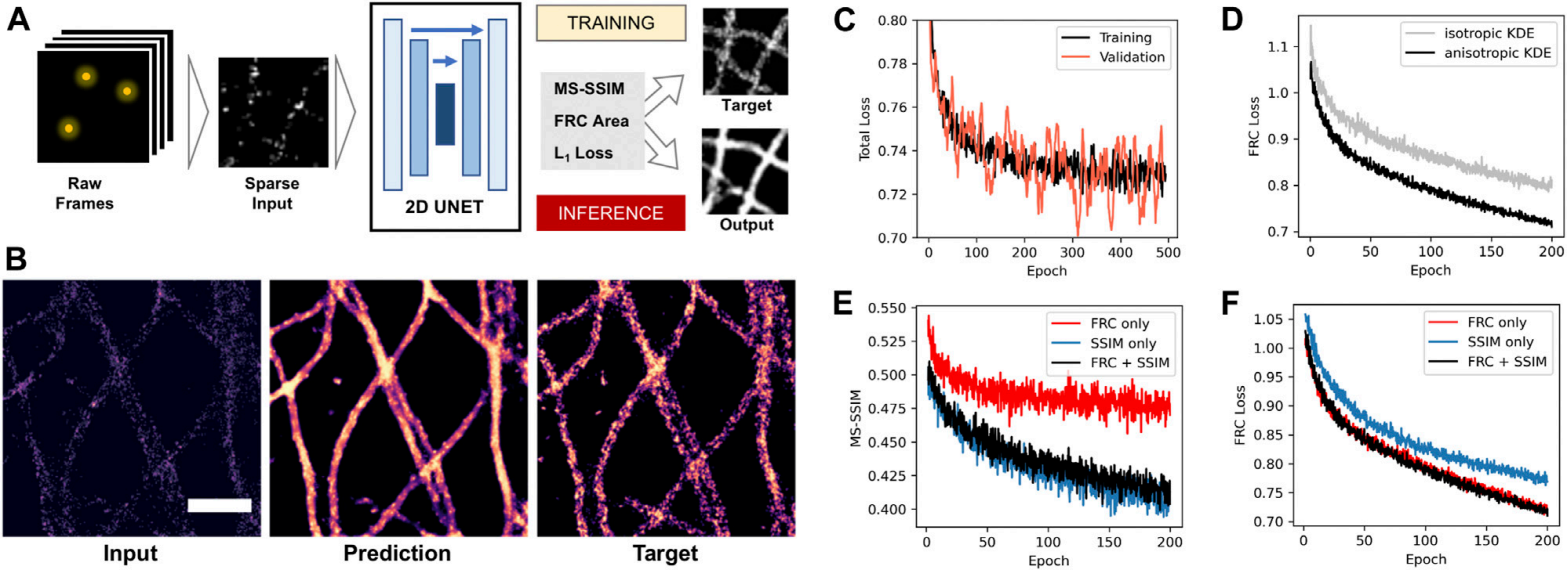
Deep learning-based density reconstruction with FRC area loss. **(A)** Deep learning workflow using a 2D U-Net architecture to predict density images from sparse input. **(B)** Example of sparse input with 10% of localizations (left), network prediction after training (middle), and target image using the full dataset (right). Scale bar = 0.5 µm **(C)** Total loss monitored during network training for training (black) and unseen validation images (orange). **(D)** FRC loss during training for target density images generated by classical isotropic Gaussian (grey) and anisotropic kernel density estimation (black). **(E)** Structural similarity index (MS-SSIM) during training for a network trained on FRC loss only (red), SSIM (blue) and both together (black). **(F)** FRC area loss during training for a network trained on FRC loss only (red), SSIM (blue), and both together (black).

We implemented our area FRC measure as differentiable function in Tensorflow2 to be able to use it as loss function for deep neural network training. We generated a set of training and validation images from a *d*STORM experiment on labelled tubulin in cells, as described in the methods section, and trained a 2D U-Net-like image-to-image fully convolutional network (Figures 3A–C) using different loss functions and targets. The final trained network was then used to predict test images not used during training to assess network performance. The evaluation criteria were peak signal-to-noise ratio (PSNR), mean squared error (MSE) or L_2_ loss, multiscale structural similarity index (MSSIM), and area FRC, independent of the loss function used during training.

We first investigated if using the anisotropic kernel density estimate as training target improves the training process. We trained the same network using MSSIM loss using either Gaussian KDE or anisotropic KDE target images and monitored the improvement of the loss during training (Figure 3D). The anisotropic density estimation target results in faster convergence of the training process. This shows that the smoothing effect of the anisotropic kernel provides a better optimization target compared to regular isotropic Gaussian density estimation for anisotropic structures like filaments.

Next, we compared our FRC area loss to MSSIM, which is the state-of-the-art loss function for image-to-image tasks and was shown to work well for SMLM sparse-to-dense reconstruction (Ouyang et al., 2018). We trained the network on the same data using either MSSIM only, FRC only, or both together, and monitored both losses during training (Figures 3E,F). We observed that the FRC loss decreases at the same rate when optimizing for FRC area only or for MSSIM + FRC, but at a slower rate when optimizing for MSSIM only. Correspondingly, MSSIM goes down at the same rate when optimizing for FRC + MSSIM as when optimizing for MSSIM only, but at a slower rate when optimizing for FRC only. This shows that there is no trade-off between both losses: using MSSIM and FRC together gives an improvement over using only one of the two. This shows that our new area FRC loss provides an improvement over using only MSSIM when both are combined.

### Quantification of Reconstruction Quality

**FIGURE 4:**
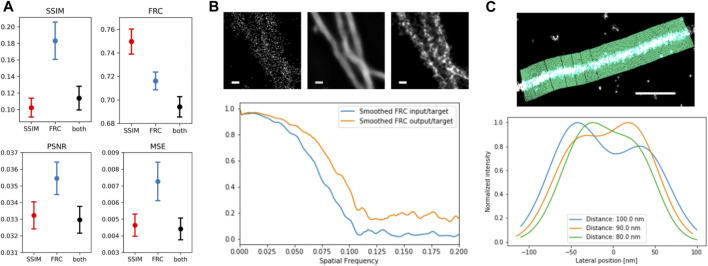
Evaluation of trained networks. **(A)** averaged MS-SIM and FRC (top), as well as peak signal-to-noise ratio (PSNR) and mean squared error (MSE) for 20 network output and target images from the validation dataset; error bars denote SEM. **(B)** FRC improvement as function of spatial frequency of input (left) vs. target (right) and output (center) vs. target. Scale bar = 100 nm. **(C)** Line Profiler evaluation illustrated at the top and applied to two adjacent simulated filaments with distance of 80 nm (green), 90 nm (yellow) and 100 nm (blue), showing the emergence of two separate peaks.

The final trained networks were used to predict images not used during training to assess network performance. The evaluation criteria were peak signal-to-noise ratio (PSNR), mean squared error (MSE) or L_2_ loss, multiscale structural similarity index (MSSIM), and area FRC, independent of the loss function used during training (Figure 4A). In all cases, the network trained on the combination of both loss functions shows comparable or improved performance. The change of the FRC as function of frequency gives information on how different length scales or frequencies contribute to the improvement (Figure 4B). The FRC of predicted and ground truth image shows a shift to higher frequencies as well as a constant offset in the frequencies above the cut-off frequency in comparison to the FRC between input and target image. The quality of reconstructed filaments and image resolution is often measured by single line profiles perpendicular to filament direction, but this criterion is not objective. The tool LineProfiler was developed to provide an unbiased measure for filament image quality (Zwettler et al., 2020). We applied LineProfiler to reconstructions of simulated sparse microtubule filament images with predefined distances (Figure 4C). By defining the ability for resolving two filaments via the existence of two peaks and a minimum, and evaluation of 20 independent simulations, we obtained a resolution capability of 89.15 ± 2.61 nm.

## Discussion

We introduced a new loss function based on the area of the FRC for deep learning-based reconstruction of SMLM density estimates for microtubules from small subsets of localizations. Preprocessing of ground truth images by a novel anisotropic kernel density estimate improved the training process. The FRC loss ideally complements the multiscale structural similarity index (MSSIM) and leads to an improved reconstruction outcome. We implemented the adaptive anisotropic KDE proposed in (Chen et al., 2014) in pixel space using a defined support window and scale to calculate the covariance matrix. While being more efficient, the disadvantage of such an image-based implementation is the fixed window size, whereas in a localization-based algorithm also far away localizations would contribute to sparse regions, limiting the influence of isolated localizations. The principle behind anisotropic KDE calculated in image space is similar to anisotropic diffusion filtering, a widely used concept in image processing (Weickert 1996).

We used the area of the FRC, as described in (Preusser et al., 2021), up to a limit of 0.2 of the maximum frequency. Using the entire FRC area, the network learned to achieve correlation at high frequencies by blurring the image, but this did not improve image quality. Fixed FRC cut-off values like 1/7 (Robert P. J. Nieuwenhuizen et al., 2013) are problematic, as discussed in (Heel et al., 2005). When used as optimization target, the resulting FRC values are sometimes just above the threshold, leading to poor image quality and high background intensity. In general, FRC as image resolution metric must be used carefully since it can give biased results (Johnson et al., 2021).

As with many deep learning-based methods, the question is how much the generated images can be trusted, or if the network makes up information that is not in the original data. In principle, the information that is lost by removing a large fraction of the localizations cannot be regained, neither by applying deep learning nor by other reconstruction methods. In other words, there is no way to infer the precise location of emitters that were either never detected, or removed from the dataset. Instead, the idea behind density reconstruction from sparse localization data is to estimate the underlying density from a small sample of emitter positions. SMLM imaging in fact always involves such an estimate rather than measuring the true emitter density, since the latter would require perfect labeling efficiency and infinite measuring time. The benefit of density reconstruction by deep learning or other means is that it can use inherent redundancy in the localization data, thus reducing the number of required localizations while only minimally compromising the reconstruction quality. One could also argue that density estimation from localization point cloud data can be seen more as a segmentation task rather than denoising or deconvolution.

Although we demonstrate our approach only on microtubule filaments, the area-FRC loss is generally applicable, since FRC works also for other structures than filaments. The feasibility of NN-based SMLM reconstruction for a variety of structures was already demonstrated in (Ouyang et al., 2018). The absolute values of the FRC are however highly dependent on the frequency content of an image and thus on the imaged structures (Heel et al., 2005; Legant et al., 2016). Images with filaments (e.g., microtubules) yield a different FRC area or “resolution” compared to more continuous structures such as mitochondria, even when imaged with the same optical resolution. Nevertheless, using FRC area at low spatial frequencies as optimization target for improving the same image is possible, since only the change of the loss measure but not its absolute value is used as criterion.

For density reconstruction from sparse localization data, ANNA-PALM presented by Ouyang et al., (2018) presents the current state-of-the-art based on conditional generative adversarial networks, or cGANs. The original authors of the cGAN architecture (Isola et al., 2017) argue that cGANs are not suitable for image segmentation as they tend to hallucinate realistic-looking details to fool the discriminator. Ouyang et al. elegantly solve this problem by using a plausibility criterion where the consistency of the restored image with respect to a widefield low-resolution image is determined. Here, we did not use a cGAN, but a simpler architecture using only a generator U-Net, and focus on comparing the performance of different loss functions and preprocessing methods. We thus see this work as complementary to ANNA-PALM, and as basis for future extensions using new architectures. For example, the standard convolutional architecture could be modified to incorporate prior knowledge about the physical constraints of the measurement process. Introducing loss functions in Fourier space has recently been shown to make deep learning-based image reconstruction and perceptual superrsolution more efficient (Fuoli et al., 2021), and might have other interesting applications in the future. We make the python code of our training workflow and our implementation of the area FRC loss and the anisotropic kernel density estimation freely available to the community so that it can easily be integrated into other deep learning workflows.

## Data Availability

The datasets presented in this study can be found in online repositories. The names of the repository/repositories and accession number(s) can be found below: https://github.com/CIA-CCTB/FRCnet.
